# Quinoline Derivatives: Promising Antioxidants with Neuroprotective Potential

**DOI:** 10.3390/antiox12101853

**Published:** 2023-10-12

**Authors:** Luis Felipe Hernández-Ayala, Eduardo Gabriel Guzmán-López, Annia Galano

**Affiliations:** Departamento de Química, Universidad Autónoma Metropolitana-Iztapalapa, Av. Ferrocarril San Rafael Atlixco 186, Col. Leyes de Reforma 1A Sección, Alcaldía Iztapalapa, México City 09310, Mexico; hdz.ayala@quimica.unam.mx (L.F.H.-A.); eggl@ciencias.unam.mx (E.G.G.-L.)

**Keywords:** rational design, quinoline derivatives, antioxidants, neuroprotection, Alzheimer and Parkinson diseases

## Abstract

Quinoline has been proposed as a privileged molecular framework in medicinal chemistry. Although by itself it has very few applications, its derivatives have diverse biological activities. In this work, 8536 quinoline derivatives, strategically designed using the CADMA-Chem protocol, are presented. This large chemical space was sampled, analyzed and reduced using selection and elimination scores that combine their properties of bioavailability, toxicity and manufacturability. After applying several filters, 25 derivatives were selected to investigate their acid–base, antioxidant and neuroprotective properties. The antioxidant activity was predicted based on the ionization potential and bond dissociation energies, parameters directly related to the transfer of hydrogen atoms and of a single electron, respectively. These two mechanisms are typically involved in the radical scavenging processes. The antioxidant efficiency was compared with reference compounds, and the most promising antioxidants were found to be more efficient than Trolox but less efficient than ascorbate. In addition, based on molecular docking simulations, some derivatives are expected to act as inhibitors of catechol-O methyltransferase (COMT), acetylcholinesterase (AChE) and monoamine oxidase type B (MAO-B) enzymes. Some structural insights about the compounds were found to enhance or decrease the neuroprotection activity. Based on the results, four quinoline derivatives are proposed as candidates to act as multifunctional antioxidants against Alzheimer’s (AD) and Parkinson’s (PD) diseases.

## 1. Introduction

Under oxidative stress (OS) conditions, there is an excess of pro-oxidants that cannot be counteracted by the antioxidant systems [1]. This triggers a state of chronic OS where cellular metabolism increases the production of free radicals in general and reactive oxygen species (ROS) in particular [2,3,4,5]. The brain consumes large amounts of oxygen to carry out its physiological functions, which promotes the production of free radicals. Some factors make the brain susceptible to ROS attack. For example, the lack of antioxidant mechanisms, its particularly rich fatty acid composition [6], and the low permeability of the blood–brain barrier [7,8,9], which reduces the passage of many antioxidants such as vitamin E. Consequently, OS has been associated with neurodegenerative diseases such as Alzheimer’s (AD) and Parkinson’s (PD) diseases, among others [10,11]. OS has been observed in these diseases, even at early stages, indicating that ROS and other free radicals could be related to their etiology [12,13].

Several lines of research have implicated OS and free radical damage in the origin and pathogenesis of AD [12,14,15,16,17]. This damage includes energy metabolism and overcompensation of antioxidant enzymes [17]. Despite the extensive literature regarding OS in AD, the causes of the increased free radical amounts that initiate such damage remain unclear. However, some candidates could be: activated microglia [18], ß-amyloid protein deposits [19], phospholipid peroxidation and protein oxidation [20], and relative high concentration of metals, such as iron and copper [21,22,23,24,25]. In the case of PD, evidence suggests that deficiencies in mitochondrial function, increased OS, apoptosis and inflammation [26,27,28,29] are part of the processes that eventually result in neurodegeneration. In addition, the ROS generated by the oxidation of dopamine have been implicated in the destruction of neurons related to aging and other neurodegenerative processes such as PD [30,31,32]. Two enzymes are involved in the dopamine oxidation process, monoamine oxidase (MAO) and catechol-O-methyl transferase (COMT), which yield considerable quantities of superoxide and hydroxyl radicals, as well as hydrogen peroxide [33,34,35].

Quinolines are aromatic heterocycles formed by the fusion of a benzene and a pyridine ring. Quinoline itself has few applications, but it is considered a privileged structure [36,37,38,39,40] from which derivatives are built that are useful in various fields [41,42,43,44], mainly in medicinal chemistry. A prominent example of these derivatives is quinine, an alkaloid found in plants that has long been the main choice in the treatment of malaria [45]. More than 200 biologically active quinoline alkaloids have been identified [46]. The primary use of this compound is as a precursor of 8-hydroxyquinoline, a versatile chelating agent and pesticide precursor [47]. The interest in studying quinoline derivatives has increased since they are of great importance for the pharmaceutical industry. This interest has driven the development of simple and eco-friendly synthesis methods that represent an advantage over other molecular scaffolds [48,49,50,51,52].

**Scheme 1:**
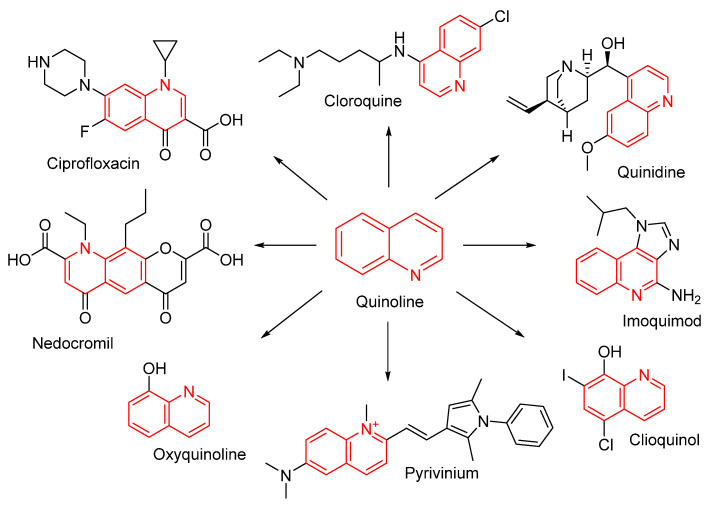
Quinoline approved drugs. Red color highlights the quinoline framework.

The abundant literature on the synthesis of quinoline and its derivatives has encouraged researchers to explore this molecular framework to develop potential drugs. Quinoline structural motif is present in many drugs used for the treatment of various diseases, mainly as antimalarial drugs [18]. Since heterocyclic molecules are used for drug discovery and development, the quinoline ring is a framework with several advantages and a wide variety of potential benefits. Its derivatives have been studied as possible antibacterial, antifungal, antimycobacterial, antiviral, antiprotozoal, antimalarial, anticarcinogenic, antioxidant, anticonvulsant, analgesic, anti-inflammatory, anthelmintic and cardio protector agents, as well as being beneficial against diseases affecting the nervous system [53,54,55,56,57,58,59,60,61,62,63,64,65,66,67,68,69,70]. Scheme 1 presents some quinoline derivatives that are approved drugs.

The systems that contain quinoline moieties are used to treat various conditions. Among the approved drugs, the major occurrences of quinoline derivatives are as antimalarial agents [53]. In this class, the first discovered drug was cloroquine [53]. These compounds are also widely used against bacterial infections [71], with ciprofloxacin being one of the most widely used. There are antispasmodic drugs, such as nedocromil; antiasthmatic (oxyquinoline); anthelmintic (pyrvinium salts), antiarrhythmics (quinidine) and dermatological treatments with imoquimod [37,38,39,40]. There are also anticancer, antiviral, antioxidant, and antipsychotic medications, among many others, that are quinoline-based compounds [72,73,74]. It is for all these reasons that some authors have called quinoline a perpetual and multipurpose scaffold in medicinal chemistry [40].

In this work, a strategical, systematic and rational search for quinoline derivatives with antioxidant and neuroprotective activities has been performed using the CADMA-Chem protocol [75]. Bioavailability, toxicity, synthetic availability, electron and hydrogen atom donating capabilities, and the potential for inhibiting COMT, AChE and MAO-B enzymes were explored. According to the obtained results, the most promising candidates were identified and proposed for further investigations.

## 2. Materials and Methods

### 2.1. Construction of Derivatives and Estimation of Molecular Properties

**Scheme 2:**
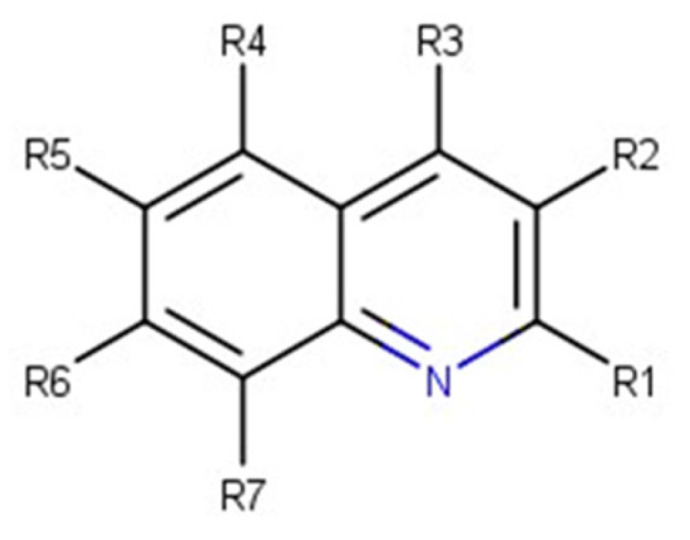
Quinoline (R1–R7=H) and their derivatives (dQ). Blue color represents the nitrogen atom.

Quinoline (Q1, Scheme 2) derivatives were systematically designed. They were built using the Smile-it tool, developed in our research group. It is available at https://agalano.com/Smile-It/ (accessed on 15 September 2023). Seven sites of the scaffold were substituted with six functional groups (–OH, –NH_2_, –SH, –COH, –COCH_3_ and –COOCH_3_). The mono, di and trisubstituted compounds were analyzed. According to this, 8356 quinoline derivatives (dQ) were designed.

For all the designed derivatives, the parameters of absorption, distribution, metabolism and excretion (ADME) were estimated with the open source chemoinformatics tools RDKit [76]. These parameters were used to confirm whether the derivatives satisfy the rules of Lipinski, Ghose, Veber, Egan and Muegge [74,75,76,77,78,79,80,81]. Compounds that violate more than one of these rules have difficulties with bioavailability and could present permeation problems. Their manufacturability was evaluated by means of the synthetic accessibility (SA) parameter calculated with the AMBIT-SA, version 3.1.0, software specialized in organic molecules [82]. A value between 0 and 100 is estimated. The higher the value, the easier the compound is to synthesize. The safety of the compounds was estimated by four descriptors, using the Toxicity Estimation Computer Tool (T.E.S.T.), version 5.1.2 [83]. Rodent median lethal dose (LD_50_), Ames mutagenicity (M), developmental toxicity (DT) and bioaccumulation factor (BF) were used to assess the toxicity of quinoline and its derivatives. The significance of each parameter and the criteria used are defined in Table S1. The complete set of calculated ADME, Toxicity and SA parameters can be consulted in an open access repository: https://github.com/luckhdz/Quinolinas/blob/main/dQ%20properties.xlsx (accessed on 11 September 2023).

To select samples from the constructed chemical space, selection and elimination scores, expressed in terms of the parameters described above, were used [84,85]. For comparison purposes, a set of reference molecules was used, which have been used as neuroprotectors or are being investigated in advanced clinical phases in this context (Table S2).

### 2.2. DFT Calculations

Electronic structure calculations were performed with the density functional theory (DFT) using Gaussian 16 [86] software. Geometric optimizations and frequency calculations were performed using the M05-2X/6-311+G(d,p) level of theory. No imaginary frequencies were obtained, i.e., the structures are minima on the potential energy surface. Solvation effects were simulated using the universal solvation model (SMD) [87], using water as solvent. M05-2X has been tested against various databases and its reliability has been validated. The tests include barrier heights, conformational energy and trends in bond dissociation energies [88]. It is also recommended to model open shell systems [88]. This function has been successfully used to estimate bond dissociation energies (BDE) and the free radical scavenging capacity of several antioxidants [89,90,91,92,93].

The ΔSCF approach [94] was used to calculate ionization energies (IE). For the estimation of BDE, all the probable sites for H donation were considered. That is, the –CH_3_ in the quinoline ester fraction, and the phenolic OH in the different functionalization sites, from R1 to R7 (Scheme 1).

Deprotonation routes were predicted with the Marvin suite [95], and the pKa values were refined with the fitted parameters procedure [96]. This property is of crucial importance for medical drugs since it governs the proportion of neutral species at a particular pH. These are the species most likely to passively cross biological barriers. This method of predicting pKa values has been tested before, with results that are close to those experimentally measured [97].

### 2.3. Protein–Ligand Docking Details

The enzyme structures were obtained from the protein data bank https://www.rcsb.org/ (accessed on 1 July 2023). The data are summarized in Table 1.

AChE misplaced loop regions (256–261 and 493–496 residues) were fixed using Modeller [101]. Water and solvent molecules, chloride ions and non-relevant species were removed with Autodock Tools [102]. Protein ionizable residues were considered at physiological pH, i.e., the protonation state of lateral chains for D, E were considered as deprotonated species and R, K and H as protonated amino acids. For quinolines, natural substrates and inhibitors atomic charges were estimated by the NBO approach at M05-2X/6-311+G(d,p) level. Docking simulations were carried out using AutoDock Vina 1.2.0 software [103]. A gradient optimization algorithm was performed inside the active site centered at x: −13.50, y: 37.69, z: 61.63 and grid size of 15 × 15 × 15 Å3 for COMT, x: 51.81, y: 156.34, z: 28.15 and grid size of 15 × 15 × 15 Å3 for MAO-B and x: −16.30, y: −43.83, z: 30.17 and grid size of 21 × 21 × 21 Å3 for AChE. Docking scores (ΔG_B_) were reported for the best docked pose and then this score was weighted (ΔG^W^_B_) according to the fractions of each relevant species at pH = 7.4. For the five most stable complexes, the conformation protein–ligand was analyzed and plotted with Discovery Studio software, version 2021 [104]. Redocking simulations were carried out. The RMSD values for inhibitors 1.8, 1.6 and 2.8 Å and the scores 7.6, 10.0 and 12.0 kcal/mol were founded for Tolcapone, safinamide and donepezil, respectively. They agree with experimental IC_50_, Ki or ΔG_B_ values [99,100,105]. These results confirm the suitability of our docking methodology.

## 3. Results and Discussion

### 3.1. Screening the Chemical Space—Selection and Elimination Scores

Due to the inclusion of –OH, –NH_2_, –SH, –COH, –COCH_3_ and –COOCH_3_ groups in the R1 to R7 sites, considering mono-, di- and trisubstitutions, 8359 quinoline derivatives were built. Of these, 42 are monosubstituted compounds, 756 are disubstituted and 7560 are trisubstituted compounds. For 2033 of them, some toxicity values could not be estimated. Thus, they were eliminated in a first screening. The calculated data for all the designed compounds can be consulted in https://github.com/luckhdz/Quinolinas/blob/main/dQ_table.pdf (accessed on 11 September 2023).

The selection score (S^S^) was the indicator applied to sample the generated chemical space. The values obtained for this selection parameter range from 2.36 to 3.54. The selection score considers the bioavailability of the compounds through the estimation of the ADME properties, toxicity and the ease with which the compound could be synthesized. According to the obtained values, the derivatives that beat the performance of the parent molecule (S^S^ = 2.83) and the average of the reference set (S^S^ = 3.00) are five hundred and thirty compounds.

This scoring system considers the averages of the variables (ADME, toxicity and synthetic accessibility) and may mask flaws in some properties. To overcome this obstacle, an elimination score (S^E^) was developed that indicates the deviations of the values of each property with respect to the reference set. This function can be analyzed as a whole or by each individual property. Furthermore, S^E^ works as an additional filter to choose the most promising derivatives. The elimination function is based on the deviation (of each property) and is a sum of the individual elimination coefficients. The details of the S^S^ and S^E^ calculations can be found in the Supporting Information.

**Scheme 3:**
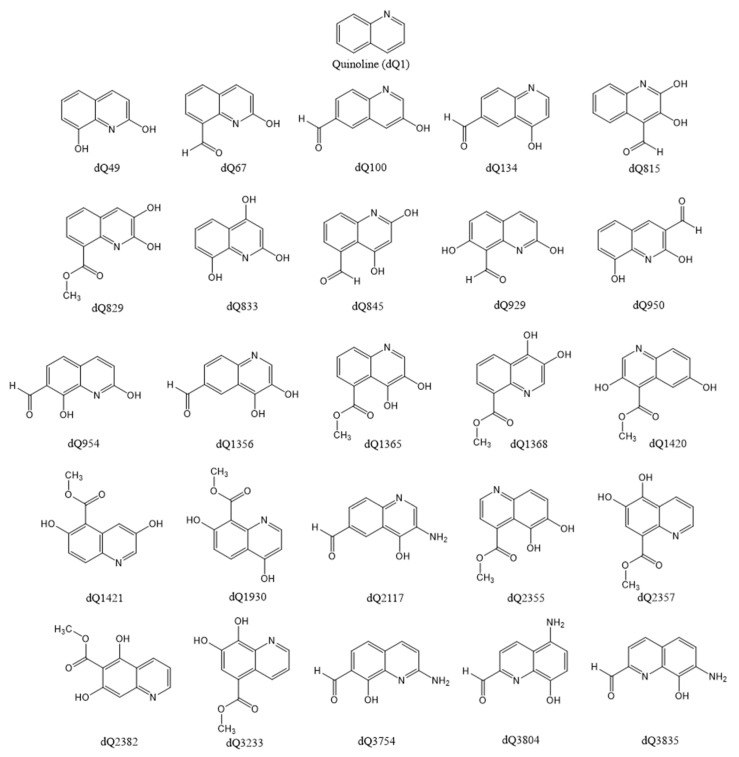
Two-dimensional structures of the best-scored (S^S^ y S^E^) quinoline derivatives.

Using this exclusion score, further evaluation was achieved. At this point, 25 derivatives were chosen. The structures of the best-scoring derivatives and the molecular framework (dQ1) are presented in Scheme 3.

**Figure 1:**
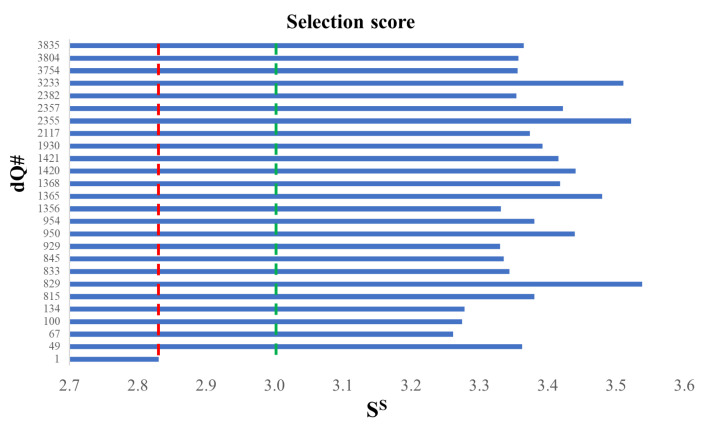
Selection score S^S^ for the best quinoline derivatives. Red line is the estimated value for quinoline scaffold and green line represents the average selection score for the reference set.

**Figure 2:**
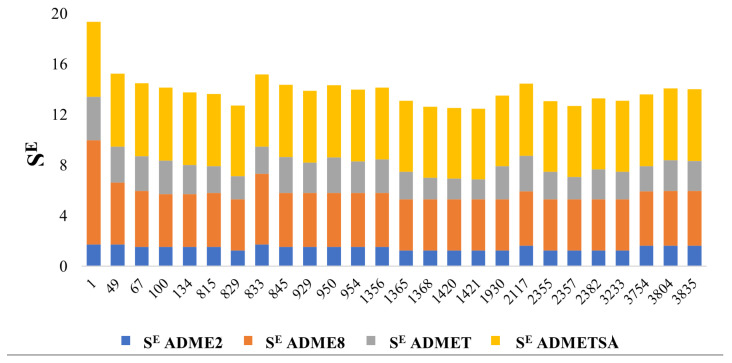
Elimination score (S^E^) by set of properties for the most promising quinoline derivatives. The size of the segments represents deviations from the estimated values of the reference set.

Due to the large number of studied compounds, the S^S^ and S^E^ plots (Figure 1 and Figure 2, respectively) are presented only for the twenty-five most promising derivatives.

**Figure 3:**
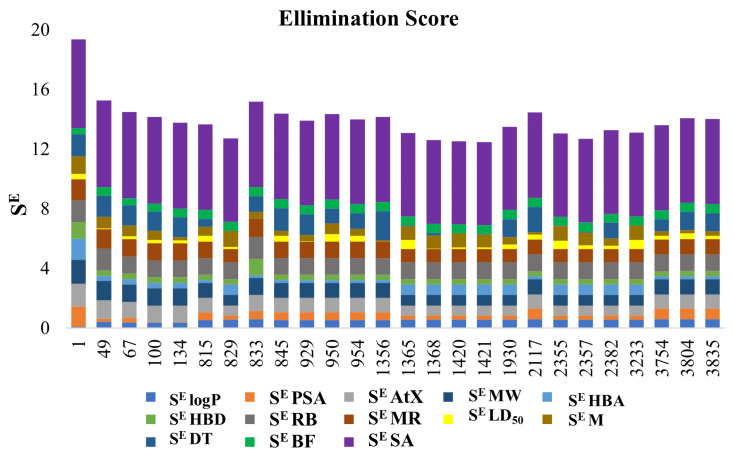
Individual contributions to the elimination score (S^E^) for the most promising quinoline derivatives.

In general, molecules with higher S^S^ values are expected to have lower toxicity, easier synthesis and better bioavailability, i.e., they would have all the desirable aspects for an oral drug. In the case of S^E^, the values range from 12.5 to 19.4, the highest value being that obtained for the parent molecule. Figure 2 presents the plot of S^E^ values for a set of properties, SADME2, SADME8, SADMET and SADMETSA. These plots show that the largest deviations (with respect to the reference set) are the ADME properties and the synthetic accessibility. The ADME properties present negative variations since the derivatives are significantly smaller than the reference set of molecules. On the other hand, the synthetic accessibility of our derivatives is an advantage over the reference neuroprotectors. Finally, this plot is not enough to analyze the deviations of toxicity parameters in detail. To that purpose, individual contribution plots are presented in Figure 3.

Except for quinoline, all the derivatives present a slightly higher LD_50_ (yellow segment). The same occurs with mutagenicity (gold segment), but the deviations are larger. For all the studied derivatives, the bioaccumulation factor (light green bar) is lower than the average of the reference set. However, this parameter shows a very large dispersion, which makes this elimination score small. The most significant case is the developmental toxicity (aqua bar). Quinoline and its derivatives are less toxic than the reference set; in some cases, the deviation is remarkable (dq845, dQ1356, and dQ2117, for example). Since the twenty-five compounds have acceptable bioavailability (no violations of the Lopinski, Egan, Muegge, Ghose and Veber rules), low toxicity and easier manufacturability (than the reference set), all compounds were kept in the next stage of the investigation to evaluate their potential antioxidant and neuroprotective activities.

### 3.2. Acid–Base Equilibria and Antioxidant Activity

For potential drugs, the study of the acid–base behavior is crucial to find out if these molecules can cross biological barriers by passive diffusion. Deprotonation pathways and estimated pKa values are shown in Figure S1 (Supporting Information). Molar fractions, represented as percentage X% at biological pH, are reported in Table 2.

For most of the compounds, the neutral species (X%dQ) predominates at pH = 7.4. However, there are several derivatives that present important anionic fractions (X%H-1dQ-). In the cases of dQ833 and dQ2382, the cationic species (X%HdQ+) has a non-negligible percentage (>1.0%). For dQ1421, the dianion is present in small amounts (1.0%). Among the analyzed derivatives, dQ829, dQ833, dQ954, dQ1365, dQ1368, dQ1930, dQ2355 and dQ2357 have important amounts of both neutral and anionic species. According to the portion of the neutral form, these compounds should not present problems to passively cross biological barriers. Additionally, the antioxidant activity can be enriched by the presence of a significant amount of the charged species [106].

**Figure 4:**
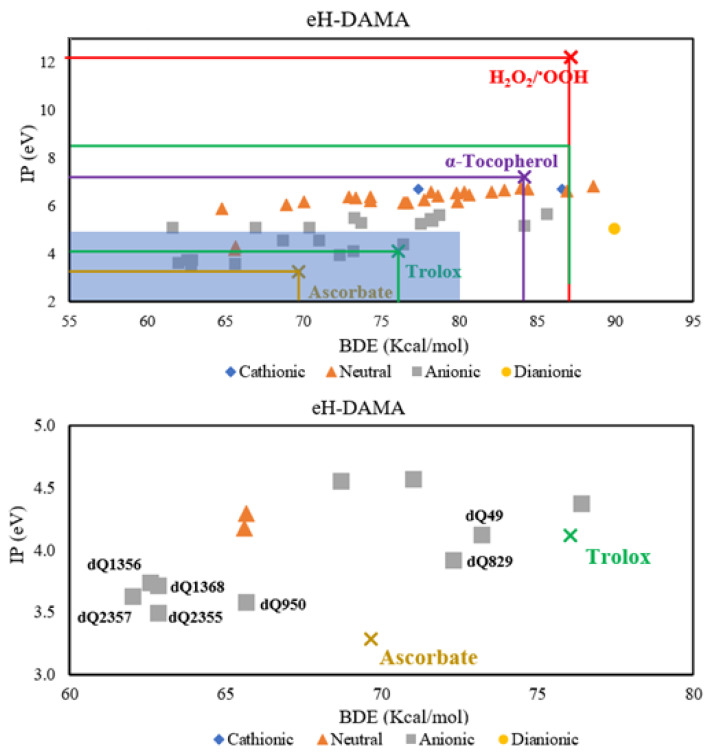
The electron and hydrogen donating ability map for antioxidants (eH-DAMA) for the most promising derivatives and their most abundant acid–base species at physiological pH (**top**). Blue box (top) represents the area that contains the most promising antioxidants. Reference antioxidants are presented for comparison purposes. Close up to the most promised antioxidants (**bottom**).

Ionization potential (IP) and bond dissociation energies (BDE) were calculated for the acid–base species with non-negligible fractions (X%dQ ≥ 1%). The results are presented in Table S3. The sp3 sites were considered as potential H-donors, i.e., phenol, amine and methyl groups, in this order. Quinoline was not considered since it does not contain this kind of site. BDE and IP are related to the capability of the compounds to donate one H-atoms and one electron, respectively. Then, these parameters were used to evaluate the efficacy of quinoline derivatives as free radical scavengers via single electron transfer (SET) and hydrogen atom transfer mechanisms (HAT), respectively. They were compared with reference antioxidants, i.e., Trolox, α-tocopherol and ascorbate. The oxidant target H_2_O_2_/^•^OOH was also included. To analyze the scavenging efficiency, the electron and hydrogen atom donation map for antioxidants (eH-DAMA) was constructed with IP and BDE (Figure 4).

According to the eH-DAMA, anionic species are the most efficient donors. Almost all compounds will be capable of scavenging hydroperoxyl radicals, and most species are predicted to be more efficient than α-tocopherol for that purpose. Derivatives dQ49, dQ829, dQ950, dQ1356, dQ1368, dQ2355 and dQ2357 were identified as the most powerful antioxidants. dQ49 has better H donor behavior than Trolox and similar efficiency as an electron donor. The results suggest that the remaining compounds would be better radical scavengers via both SET and HAT mechanisms than Trolox. Five compounds have better radical scavenging performance via the HAT mechanism than ascorbate (vit. C), but are less efficient via the SET mechanism. At this point, it is worth mentioning that vitamin C is one of the antioxidants present in the biological environment [107].

### 3.3. Neuroprotection Assessment

To assess the neuroprotective activity of quinoline derivatives, the weighted docking scores and polygenic score (S^P^) of the most promised candidates are reported in Table 3. The complete set of docking data can be found in Table S4. S^P^ is a measure of the compounds affinity towards enzymes, compared to that of their natural substrates: dopamine (COMT), phenylethylamine (MAOB), and acetylcholine (AChE). It is defined based on previous reports [93].

Based on the docking data, quinoline presents less affinity than the natural substrates of COMT and AChE, this is the origin of its lower S^P^ value. As with other properties, it seems that the molecular framework itself is of lower chemical or biological interest than its derivatives. On the other hand, the S^P^ values of the functionalized derivatives suggest that these compounds may exhibit neuroprotective activity since their score is higher than those of the natural substrates. In general, quinoline derivatives have a good affinity with enzymes, and can inhibit their natural function. Among the studied compounds, dQ929 is predicted to be the most promising neuroprotector.

**Figure 5:**
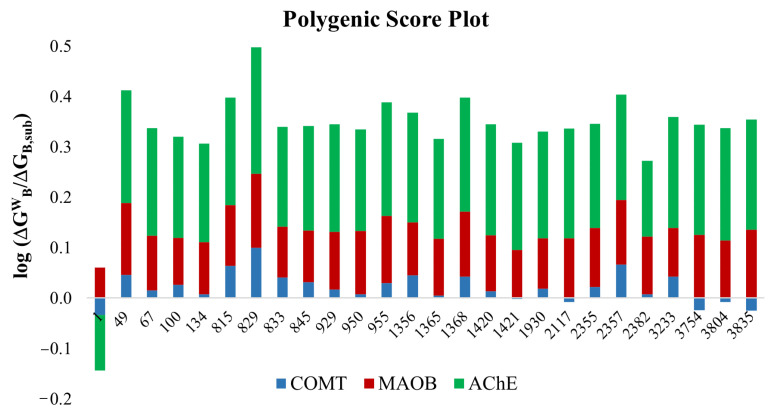
Polygenic score (SP) for the most promised quinoline derivatives presented as individual affinities.

Although most of the compounds presented enhanced S^P^ values, analysis of individual affinities can reveal interesting data on the behavior of protein–ligand complexes. To that purpose, a polygenic score plot (PSP) is presented in Figure 5. The colored fragments represent the affinity for each enzyme. They are calculated as the logarithm of the ratio of the docking scores for the derivatives and the substrate (ΔG^W^_B_/ΔG_B,sub_); the higher the bar, the greater the affinity of the compound for each enzyme. On the contrary, negative values mean a weaker binding for the derivative than for the natural substrate, i.e., it is not expected to present neuroprotective activity.

**Figure 6:**
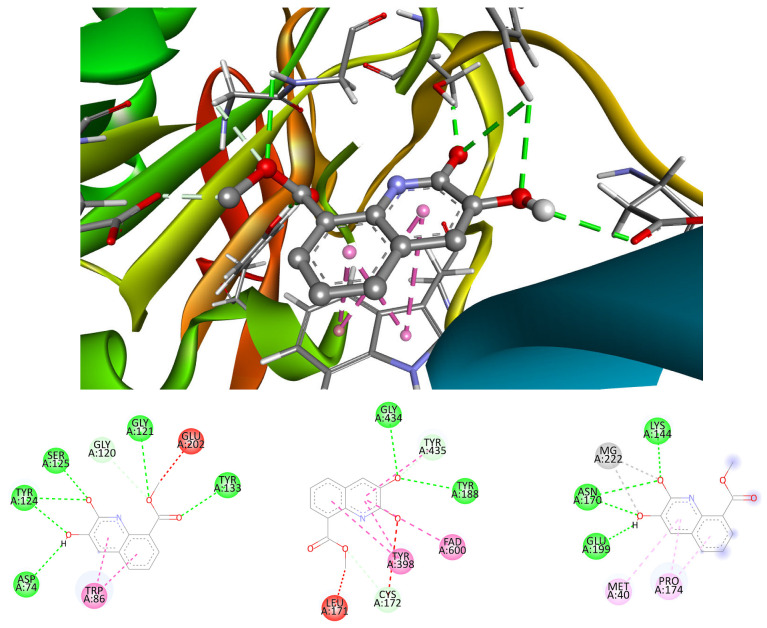
Three-dimensional representation of the interactions between dQ829 and the AChE (**top**). Bottom: Two-dimensional interaction map for the complexes of the quinoline analog with AChE (**left**), MAOB (**middle**) and COMT (**right**). H-bonds are represented in green color, non-conventional C–H bonds in light green, metal–donor in gray, π-interactions in pink and purple and steric effects in red.

According to the data detailed in Figure 6, quinoline derivatives could preferentially inhibit the action of AChE and MAOB enzymes (red and green fragments, respectively). In the case of COMT (blue fragments), the behavior of the compounds is heterogeneous. Some of them can inhibit the enzyme (dQ829 and dQ2357), while others have practically the same affinity as dopamine (dq950 and dQ1421, log = 0), and others are not likely to act as inhibitors of this enzyme (dQ2117, dQ3754, dQ3804, dQ3835). Interestingly, and contrary to what would be expected, it seems that the amino groups do not promote binding to the receptor, since all tested amino compounds presented negative values. A possible explanation for this finding is that, unlike the substrate, the amino groups of the quinoline derivatives are directly attached to the aromatic rings, whereas in dopamine this group is attached to the more accessible and flexible aliphatic chain. On the other hand, the large length of the AChE and MAOB fragments indicate a better activity against the degradation of phenylethylamine and acetylcholine, respectively. Regarding the groups that seem to favor the affinity for the enzyme, they are the aldehyde and ester carbonyl groups. Five of the six compounds with the highest affinity present these groups. In the case of AChE, the explanation could be the recognition of these groups, since this enzyme is highly specialized in the hydrolysis of esters [108]. In the case of MAOB, it would be the greatly hydrophobic environment of the active site, rich in aromatic amino acids, which would promote the stability of the complex. This would explain the activity of the molecular framework, since it is the only enzyme to which it binds strongly enough, presumably because of hydrophobic interactions.

At the top of Figure 6, a 3D diagram is presented of the stabilizing interactions in the complex formed between dQ829 and AChE. At the left bottom, a 2D map is presented that describes in a better way this interaction path. Several H-bonds are originated by the hydroxyl and carboxyl-ester groups and the residues of the protein pocket, some of them with active site key amino acids. The ester moiety is in the anionic site of the enzyme, interacting with Gly121 and Gly122 via two H-bonds involving the carbonyl oxygen. This group seems to mimic the acetylcholine binding, forming an unfavorable bump interaction, blocking catalytic site (Ser203-Glu-Hys447). On the other hand, one of the hydroxyl groups forms another H-bond with the residue Tyr124 in the peripheral site. The Trp86 in the acyl site binds via π-stacking interactions, reminding the reversible AChE inhibitor tacrine [109], which, by the way, has a similar structure to quinoline. Due to this kind of interaction, dQ829 seems to be a pseudo-reversible inhibitor of the AChE enzyme.

The 2D diagram corresponding to the interaction between MAO-B and dQ829, is presented at the bottom middle of Figure 6. The stabilization of the adduct is of multiple nature. This is reasonable considering the versatility of functional groups that this quinoline derivative contains. Some of the most important interactions involve the tyrosine fragments, and are of hydrophobic nature. These residues have a critical role in the maintenance of stable and active conformation of this enzyme [110]. Likewise, the union of dQ829 with the FAD fragment, via π-forces, would effectively inhibit the action of MAOB, since this is responsible for the oxidation of amines [111].

The interaction between the active site of COMT and dQ829 is presented at the bottom right of Figure 6. In this case, the catechol group is responsible for the stabilization of the adduct. There are several examples in the literature that indicate the inhibition of the dopamine degradation by catechol-type compounds [112]. In fact, Tolcapone is a nitro-catechol compound used as therapeutic drug against Parkinson’s disease. Interestingly, the interaction between the quinoline analog dQ829 with COMT and that of Tolcapone are very similar. Both compounds present two metal–donor interactions between the Mg(II) ion and the catechol moiety. The same oxygen atoms form H-bonds with Lys144, Asn170 and Glu199. The differences arise from the number of H-bonds that each residue can form. Tolcapone forms two of them with Glu199 (deprotonated residue), while dQ829 forms them with Asn170 (neutral residue). Another difference is the stabilization provided by the nitro group in Tolcapone that the quinoline derivative lacks. It seems to influence the stability of the formed complexes, with Tolcapone having a higher affinity for COMT than dQ829. Nevertheless, according to the results of the simulations, this functionalized quinoline is predicted to have significant neuroprotective activity. Although various catechol compounds were tested, none of them performed as well as dQ829. The origin of this remains unclear.

Docking simulations indicate that some compounds present as high performance as Tacrine in the inhibition of MAO-B and AChE. In this context, six quinoline derivatives are proposed as promising candidates against AD and anxiety disorders. According to the results related to COMT enzyme, dQ829 is a promising molecule to be investigated against PD. 

The main goal of this study was to computationally design quinoline derivatives that behave as multifunctional antioxidants with possible neuroprotective activity using the CADMA-Chem protocol. However, it seems worthwhile to mention that experimental tests are still required to validate (or not) the theoretical predictions. Hopefully, the presented results will promote further investigations on the designed derivatives proposed here as the most promising ones. The future directions of this study include the synthesis of these compounds, as well as in vitro and in vivo experiments. Regarding the protocol, its use is being expanded to search for potential therapeutic agents against other chronic degenerative diseases such as cancer and diabetes.

## 4. Conclusions

Quinoline is a privileged molecular scaffold to build derivatives with interesting biological activities. In this work, 8356 quinoline derivatives were built by adding –OH, –SH, –NH_2_, –COH, –COCH_3_ and –COOCH_3_ to the quinoline framework. These compounds were sampled and analyzed using the CADMA-Chem protocol to identify the most promising candidates as multifunctional antioxidants with neuroprotective effects.

Through the selection (S^S^) and elimination scores (S^E^), the initial set was reduced to 25 compounds, which according to the obtained results, would present less toxicity, improved bioavailability and easy manufacturability, which are desirable properties of oral drugs.

Derivatives dQ49, dQ829, dQ950, dQ1356, dQ1368, dQ2355 and dQ2357 were found to have the most promising free radical scavenging capability. Their enhanced efficiency compared to reference antioxidants (Trolox and α-tocopherol) arises from single electron transfer (SET) and hydrogen atom transfer (HAT) mechanisms. However, these compounds present less scavenging activity than ascorbate via SET, but better antioxidant behavior via HAT.

On the other hand, the coupling simulations indicate that quinoline only presents neuroprotective activity regarding the MAOB enzyme. On the contrary, some of its derivatives can act as inhibitors of this enzyme and AChE. The most efficient compounds for this purpose are dQ49, dQ815, dQ829, dQ954, dQ1368 and dQ2357. Additionally, derivative dQ829 might also inhibit the COMT enzyme by binding in a very similar way as Tolcapone, a drug used to treat PD. Some structural details that favor neuroprotective activity were found. Compounds with amino groups do not necessarily favor dopamine protection, while catechol groups enhance it to some extent. Aldehyde and ester groups improve the affinity towards AChE.

Considering the obtained data, altogether, derivatives dQ49, dQ829, dQ8368 and dQ2357 are proposed as promising molecules for further investigations. dQ829 was identified as the best candidate to act as an antioxidant with potential neuroprotective effects against Parkinson’s and Alzheimer’s diseases.

## Figures and Tables

**Table 1:** Data of proteins used in the docking simulations.

Enzyme	Co-Crystallized Inhibitor	Substrate
COMT	Tolcapone [98]	Dopamine
MAOB	Safinamide [99]	Phenylethylamine
AChE	Donopezil [100]	Acetylcholine

**Table 2:** Estimated percentage fractions of the dQ acid–base species at pH = 7.4.

dQ	*X_%_*H_2_dQ^2+^	*X_%_*HdQ^+^	*X_%_*dQ	*X_%_*H_-1_dQ^−^	*X_%_*H_-2_dQ^2−^	*X_%_* H_-3_dQ^3−^
1	---	0.0	100.0	---	---	---
49	---	0.0	79.6	29.4	0.0	---
67	---	0.0	98.7	1.3	---	---
100	---	0.0	84.9	15.1	---	---
134	---	0.0	100.0	0.0	---	---
815	---	0.0	92.4	7.6	0.0	---
829	---	0.0	30.9	69.1	0.0	---
833	---	1.1	63.3	35.6	0.0	0.0
845	---	0.0	98.7	1.3	0.0	---
929	---	0.0	88.6	11.4	0.0	---
950	---	0.0	98.8	1.2	0.0	---
954	---	0.0	59.7	40.3	0.0	---
1356	---	0.0	97.7	2.3	0.0	---
1365	---	0.0	68.1	31.2	0.0	---
1368	---	0.0	31.4	68.6	0.0	---
1420	---	0.3	28.4	71.3	0.0	---
1421	---	0.1	80.1	18.8	1.0	---
1930	---	0.0	56.8	43.2	0.0	---
2117	---	0.1	99.9	0.0	---	---
2355	---	0.0	56.3	43.7	0.0	---
2357	---	0.0	68.6	31.4	0.0	---
2382	---	4.3	92.8	2.9	---	---
3233	---	0.1	54.0	46.0	---	---
3754	---	0.0	98.0	2.0	---	---
3804	0.0	0.0	98.0	2.0	---	---
3835	0.0	0.0	98.8	1.2	---	---

**Table 3:** Scoring values of quinoline derivatives.

dQ	ΔG_B_^W^ (kcal/mol)			S^P^
	COMT	MAO-B	AChE	
Quinoline	−5.00	−6.90	−3.80	2.85
49	−6.00	−8.34	−8.20	4.18
815	−6.26	−7.91	−9.02	4.11
829	−6.80	−8.41	−8.74	4.44
954	−5.78	−8.16	−8.24	4.11
1368	−5.96	−8.07	−8.24	4.13
2357	−6.29	−8.06	−7.93	4.13

ΔG_B_, _dopamine_ = −5.4 kcal/mol in COMT; ΔG_B_, _phenylethylamine_ = −6.0 kcal/mol in MAO-B; ΔG_B_, _acetylcholine_ = 4.9 kcal/mol in AChE. For natural substrates, S^P^ = 3.00.

## Data Availability

The data presented in this study are available in the article and Supplementary Materials.

## Supplementary Materials

The following supporting information can be downloaded at: https://www.mdpi.com/article/10.3390/antiox12101853/s1, Table S1. Properties determined to the quinoline derivatives, Table S2. ADME, toxicity and synthetic accessibility of the reference set Figure S1. Deprotonation paths and pKa values for the 25 most promising dQ. Table S3. Ionization energy and bond dissociation energy of quinoline derivatives and Table S4. Complete set of docking values.
