# Supplementary material for: Quinoline Derivatives: Promising Antioxidants with Neuroprotective Potential
Source: Antioxidants (Basel). 2023 Oct 12;12(10):1853. doi: 10.3390/antiox12101853 (PMC10604020; doi:10.3390/antiox12101853)
Supplement: Supplementary file 1 [file antioxidants-12-01853-s001.zip › antioxidants-2653540-supplementary.pdf]

Quinoline Derivatives: Promising Antioxidants with Neuroprotective Potential

Luis Felipe Hernandez-Ayala, Eduardo Gabriel Guzmán-López and Annia Galano *

Supporting information

**Contents**	**Page**
**Table S1.** Properties determined to the Quinoline derivatives	**1**
**Table S2**. ADME, toxicity and synthetic accessibility of the reference set	**2**
**Figure S1.** Deprotonation paths and pka values for the 25 most promising dQ	**4**
**Table S3**. Ionization energy and bond dissociation energy of quinoline derivatives	**7**
**Table S4.** Complete set od docking values	**9**

Table S1. Properties determined to the Quinoline derivatives and selection criteria

**Abbreviation**	**Property**	**Description**
LogP	Lipophillicity coefficient	The ADME parameters were estimated using the RDKit package. If the ADME properties comply with the Lipinski, Ghose, Veber, Muggen and Egan rules, the substance is considered to have good bioavailability. A positive (1) or negative (0) value (Sprop= SLogP, SPM, etc.) is assigned if the substance meets the criteria established by the rules, as in some cases the criteria are repeated, the strictest of them is taken.
MW	Molecular weight (g/mol)	
MR	Molar refractivity (m^3/mol)	
TPSA	Topological polar surface area (Å^2)	
#At	Heavy atoms number	
HBA	H-bonds acceptor	
HBD	H-bond donnor	
RB	Rotable Bonds	
LD_{50}	Media letal doses (mg/kg)	Toxicity parameters were calculated using the TEST program. In particular, the median lethal dose is the amount of substance (mg/Kg body weight) that causes 50% of deaths in a population of rats after oral ingestion. A substance is more toxic the lower its estimated LD50 value [1].
M	Ames mutagenicity	An agent is positively mutagenic if it induces the growth of a genetically altered strain of Salmonella typhimurium, which has had its growth reverted. If $M \geq 0.5$, mutagenicity is considered positive, while if $M < 0.5$ mutagenicity is negative [2].

TD	Developmental toxicity	Whether or not a chemical causes developmental toxicity effects in humans or animals. If TD ≥ 0.5 the substance is toxic for development and if TD < 0.5 it is not [3].
BF	Bioaccumulation factor	Ratio of chemical concentration in fish because of uptake through the respiratory surface to that in steady-state water. While the value of FB is larger, the toxicity of the substance is higher [4].
SA	Synthetic accesibility	Synthetic accessibility was calculated with the Ambit-SA[R] program. This parameter indicates the ease of synthesis of a molecule. In the case of Ambit-SA, a molecule that is easy to synthesize has SA ≈ 100 and a molecule that is difficult to synthesize has SA ≈ 0.

Selection criteria of ADME properties

**Rules**	**Criteria**
**Lipinski**	HBD ≤ 5, HBA ≤ 10, MW < 500 y Log P < 5.0
**Ghose**	-0.4 ≤ LogP ≤ 5.6, 40 ≤ MR ≤ 130, 180 ≤ MW ≤ 480, 20 ≤ #At ≤ 70
**Veber**	RB ≤ 10, TPSA ≤ 140
**Muggen**	200 ≤ MW ≤ 600, -2.0 ≤ LogP ≤ 5.0, TPSA ≤ 150, RB ≤ 15, HBD ≤ 5 y HBA ≤ 10
**Egan**	-1.0 ≤ LogP ≤ 5.8, TPSA ≤ 130
**This work**	-0.4 ≤ LogP ≤ 5.0, 160 ≤ MW ≤ 480, 40 ≤ MR ≤ 130, TPSA ≤ 130, 10 ≤ #At ≤ 70, RB ≤ 15, HBD ≤ 5 and HBA ≤ 10

}

Table S2. ADME, toxicity and synthetic accessibility of the reference set

Compound	logP	PSA	AtX	MW	HBA	HBD	RB	MR	LD$_{50}$	M	DT	BF	SA
Acetylcarnitine	-1.24	66.43	14	203.24	4	0	5	48.08	N/A	N/A	0.65	4.46	89.91
Amantadine	1.91	26.02	11	151.25	1	1	0	45.09	287.44	0.18	0.75	106.10	65.15
Apomorphine	2.85	43.70	20	267.33	3	2	0	77.99	153.32	1.08	0.81	79.28	66.21
Baclofen	1.86	63.32	14	213.66	2	2	4	55.50	414.85	0.18	0.83	1.82	89.13
Benserazide	-1.76	148.07	18	257.25	7	7	5	61.48	1825.70	0.47	0.63	0.26	85.47
Benzatropine	4.42	12.47	23	307.44	2	0	4	93.41	341.43	0.21	0.62	N/A	74.54
Biperiden	3.96	23.47	23	311.47	2	1	5	94.09	347.75	0.04	0.72	341.51	73.52
Bromocriptine	3.19	118.21	43	654.61	6	3	5	164.16	58.41	0.11	1.39	N/A	4.53
Cabergoline	3.19	71.68	33	451.62	4	2	8	132.37	573.24	0.54	1.02	8.71	39.00
Carbidopa	-0.05	115.81	16	226.23	5	5	4	56.84	1996.23	0.79	0.71	0.51	79.61
Curcumin	3.37	93.06	27	368.39	6	2	8	102.02	1411.26	0.13	0.91	13.83	74.57
Dantrolene	1.74	118.05	23	314.26	6	1	4	78.64	520.79	0.55	0.84	12.64	76.26
Donepezil	4.36	38.77	28	379.50	4	0	6	110.13	716.51	0.13	0.73	53.91	72.29
Entacapone	1.78	127.70	22	305.29	6	2	5	77.94	1009.98	0.94	0.97	1.26	81.65
Galantamine	1.85	41.93	21	287.36	4	1	1	79.80	490.38	0.59	0.94	54.61	46.33
Ladostigil	2.35	41.57	20	272.35	3	1	4	78.67	215.22	0.20	0.89	15.94	69.78
L-DOPA	0.05	103.78	14	197.19	4	4	3	49.09	2624.71	0.20	0.53	0.26	81.53
Lisuride	2.84	51.37	25	338.46	2	2	3	101.64	343.15	0.71	1.09	24.46	49.96
Masitinib	5.26	73.39	36	498.66	7	2	7	146.98	2844.94	0.52	0.81	6.61	66.10
Melatonin	1.86	54.12	17	232.28	2	2	4	67.24	1913.49	0.16	0.77	3.88	78.11
Memantine	2.69	26.02	13	179.31	1	1	0	54.32	277.49	-0.29	0.70	151.48	62.45
Modafinil	2.01	60.16	19	273.36	2	1	5	76.93	2301.01	N/A	0.74	13.79	84.62
Piribedil	1.53	50.72	22	298.35	6	0	3	82.09	486.40	0.19	0.91	35.85	76.33
Pramipexole	1.58	50.94	14	211.33	4	2	3	60.63	1980.96	0.15	0.52	6.63	74.35
Procyclidine	3.94	23.47	21	287.45	2	1	5	87.20	395.47	-0.03	0.48	165.96	82.08
Remacemide	2.22	55.12	20	268.36	2	2	5	81.14	870.47	0.30	0.51	33.87	83.58
Riluzole	2.78	48.14	15	234.20	4	1	1	50.72	173.51	1.11	0.86	6.74	78.46
Rivastigmine	2.76	32.78	18	250.34	3	0	4	72.87	389.57	0.65	0.85	9.82	79.66
Ropinirole	2.85	32.34	19	260.38	2	1	7	79.50	454.08	0.34	0.61	25.42	78.40
Selegiline	2.18	3.24	14	187.29	1	0	4	61.07	311.03	1.00	0.37	38.84	83.80
Tacrine	2.70	38.91	15	198.27	2	1	0	62.80	1094.28	0.92	0.51	52.98	76.33
Tetrabenazine	3.24	38.77	23	317.43	4	0	4	90.13	554.92	0.30	0.84	109.31	69.21
Tizanidine	1.72	62.20	16	253.72	6	2	1	66.35	424.51	0.65	0.64	4.92	79.74
Tolcapone	2.55	100.67	20	273.24	5	2	3	71.04	2956.93	0.62	0.84	14.71	82.74
Trihexyphenidyl	4.33	23.47	22	301.47	2	1	5	91.82	519.12	0.09	0.59	174.69	81.82
Brexpiprazole	4.72	48.57	31	433.58	5	1	7	129.78	588.07	0.60	0.92	22.66	66.08
Levitiracetam	-0.13	63.40	12	170.21	2	1	3	44.22	2412.72	0.06	0.81	0.98	83.72
Atuzaginstat	2.86	81.42	27	386.41	4	2	10	93.61	352.37	0.38	0.78	4.26	71.62
Blarcamesine	3.53	12.47	21	281.40	2	0	4	86.28	159.46	-0.20	0.36	272.63	81.46

Caffeine	-1.03	61.82	14	194.19	6	0	0	51.20	222.13	0.05	0.80	1.26	79.68
Escitalopram	3.81	36.26	24	324.40	3	0	5	90.91	688.70	0.29	0.78	211.14	68.02
Guanfacine	1.55	78.97	15	246.10	2	3	2	60.22	845.71	0.94	0.97	3.43	87.53
Hydralazine	0.92	63.83	12	160.18	4	2	1	47.35	315.46	0.78	0.47	8.42	83.09
Eicosapentaenoic Acid	6.47	26.30	24	330.51	2	0	14	104.94	9700.47	0.81	0.54	33.15	88.40
Metformin	-1.03	88.99	9	129.17	2	4	0	36.46	156.48	0.08	N/A	N/A	93.69
Nilotinib	6.36	97.62	39	529.53	7	2	6	140.98	N/A	0.43	0.87	16.34	62.79
Simufilam	1.04	35.58	19	259.35	3	1	2	74.46	524.01	0.37	0.51	19.79	79.94
Valiltramiprosate	-0.64	109.49	15	238.31	4	3	6	56.86	1156.59	0.43	0.85	0.67	82.60
Opicapone	3.28	149.46	27	413.17	8	2	3	93.61	1772.59	0.25	0.69	67.49	74.81
Rotigotine	4.27	23.47	22	315.48	3	1	6	93.81	702.34	0.25	0.86	84.25	70.26
Rasagiline	1.90	12.03	13	171.24	1	1	2	54.34	279.24	0.20	0.85	20.60	75.60
Istradefylline	2.12	80.28	28	384.44	8	0	6	109.06	1642.55	0.49	N/A	20.61	70.05
Pimavanserin	4.67	44.81	31	427.56	3	1	8	121.47	645.60	0.10	0.80	58.30	73.81
Fingolimod	3.20	66.48	22	307.48	3	3	12	92.93	5986.27	-0.05	0.61	20.86	86.39

dQ1

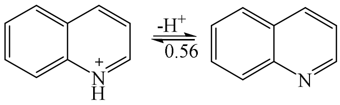

dQ49

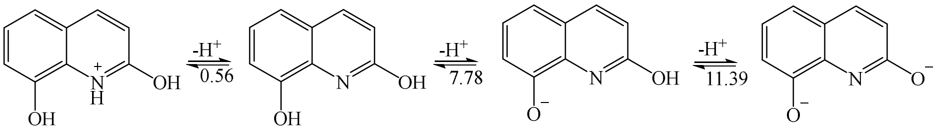

dQ67

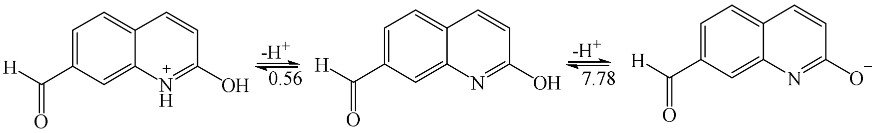

dQ100

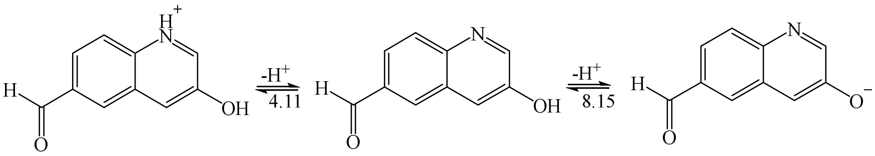

dQ134

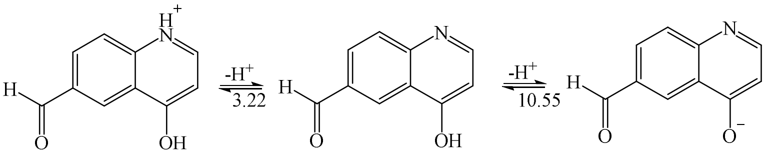

dQ815

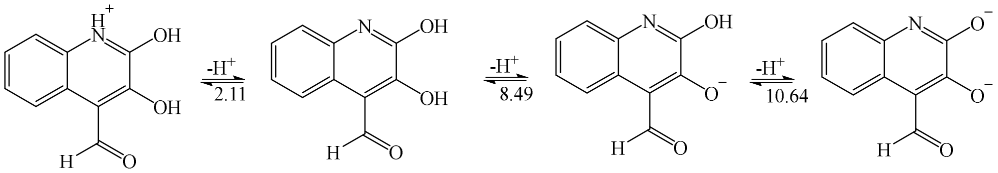

dQ829

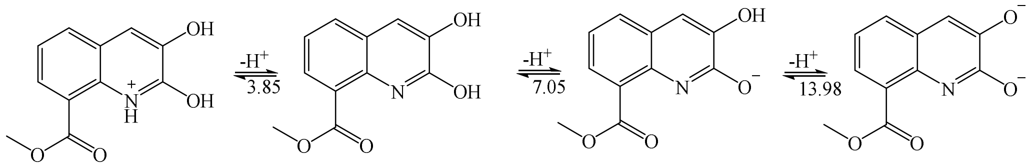

dQ833

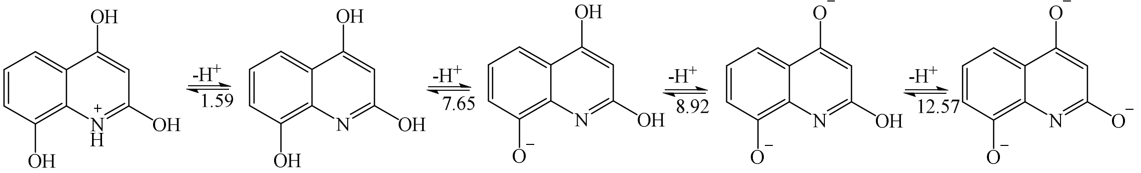

dQ845

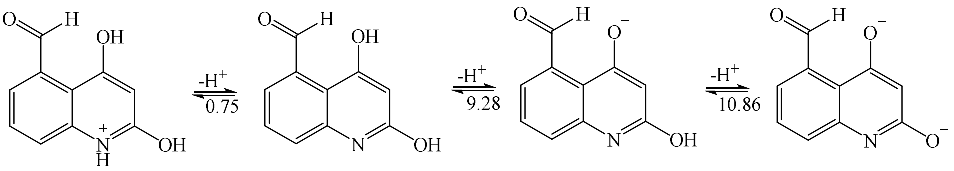

Figure S1. Deprotonation paths and pka values for the 25 most promising dQ

dQ929

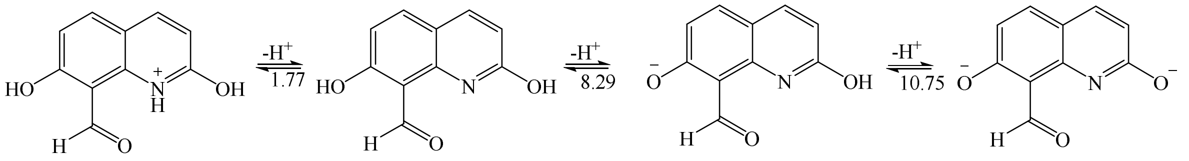

dQ950

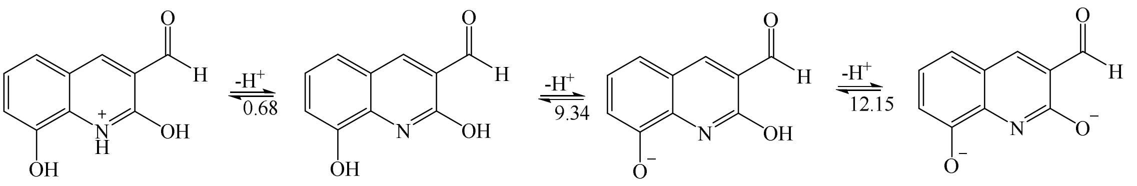

dQ954

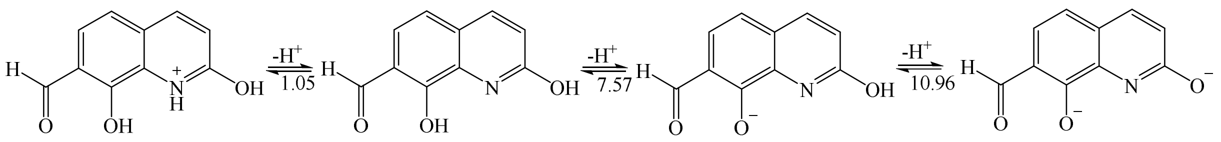

dQ1356

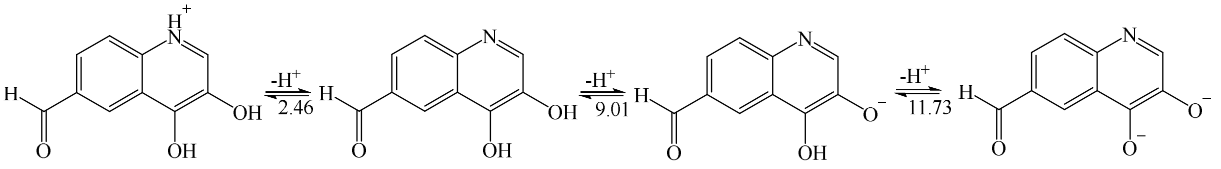

dQ1365

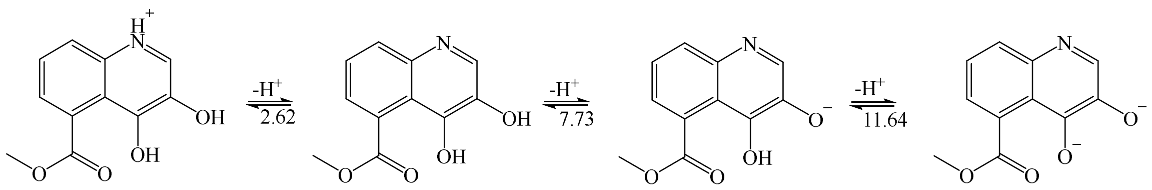

dQ1368

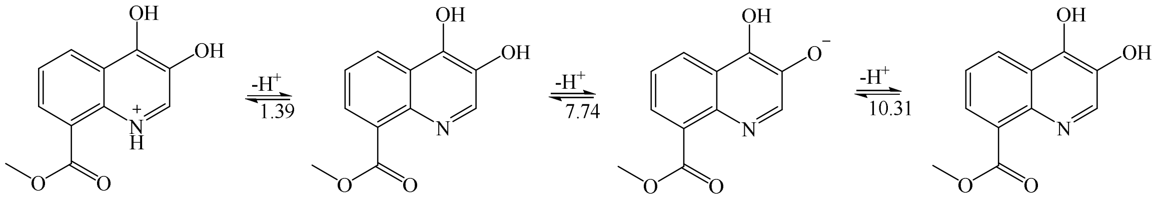

dQ1420

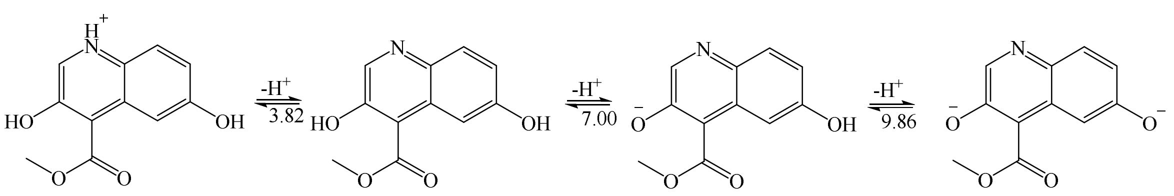

dQ1421

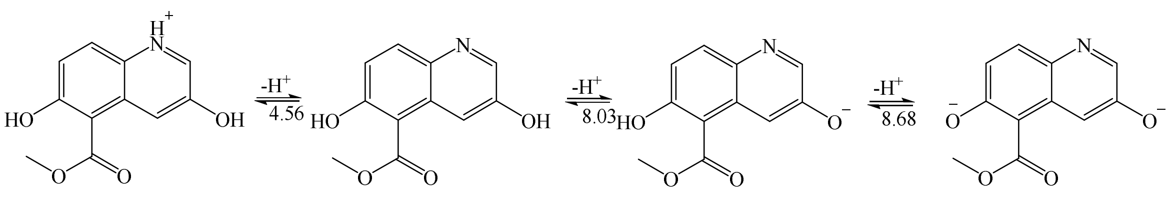

Figure S1. Cont…

dQ1930

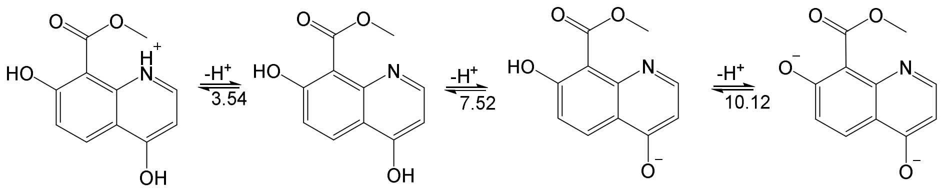

dQ2117

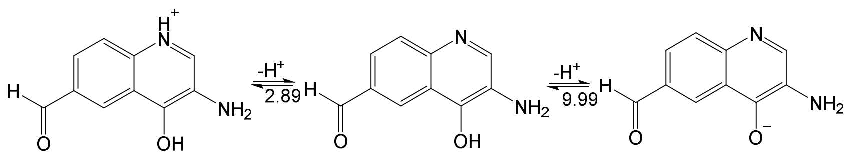

dQ2355

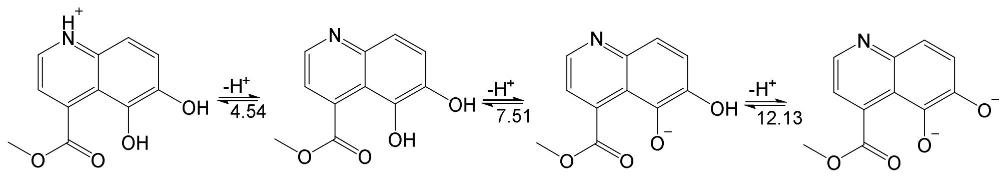

dQ2357

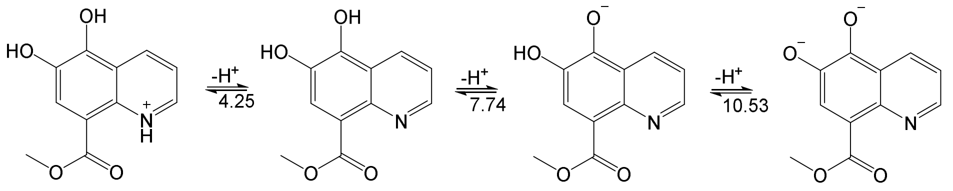

dQ2382

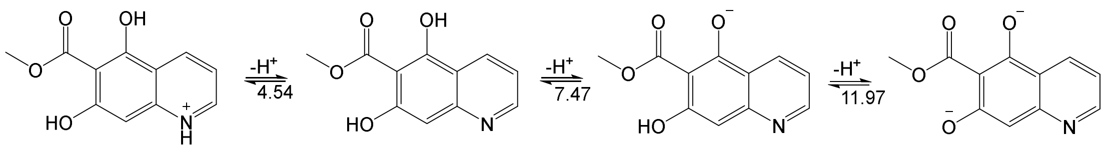

dQ3233

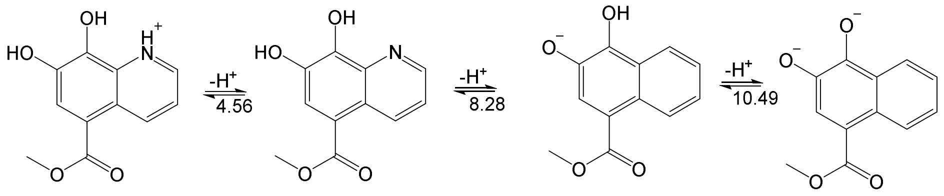

dQ3754

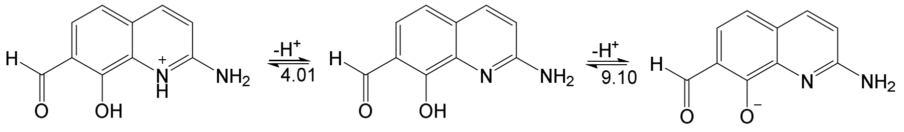

dQ3804

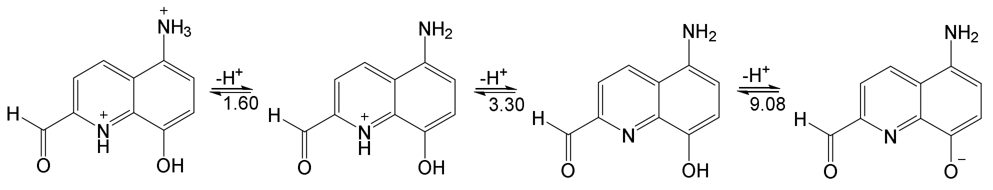

dQ3835

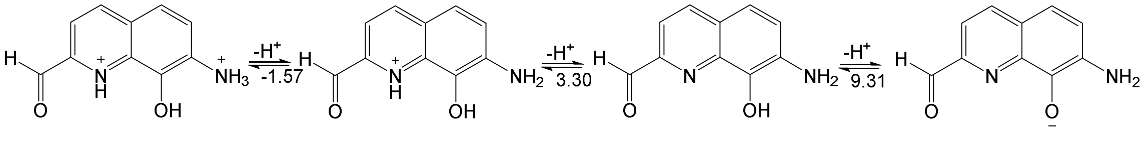

Figure S1. Cont…

Table S3. Ionization energy and bond dissociation energy of quinoline derivatives				
**dQ**	**q**	**I (eV)**	**Site**	**BDE (Kcal/mol)**
49	0	6.15	OH (R1)	76.68
			OH (R7)	83.27
	-1	4.12	OH (R1)	73.21
67	0	6.85	OH (R1)	88.55
100	0	6.60	OH (R2)	80.30
134	0	6.77	OH (R3)	83.92
815	0	6.66	OH (R2)	82.87
			OH (R1)	86.31
	-1	4.37	OH (R1)	76.42
829	0	6.57	OH (R1)	83.38
			OH (R2)	78.20
			CH_3 (ester R7)	90.41
	-1	3.92	OH (R2)	72.32
			CH_3 (ester R7)	90.45
833	1	6.69	OH (R7)	77.38
			OH (R3)	82.32
			OH (R1)	89.74
	0	6.12	OH (R7)	82.37
			OH (R3)	76.40
			OH (R1)	82.88
	-1	5.48	OH (R3)	73.30
			OH (R1)	78.48
845	0	6.70	OH (R3)	84.38
			OH (R1)	88.47
	-1	5.65	OH (R1)	85.62
929	0	6.64	OH (R6)	88.74
			OH (R1)	86.90
	-1	5.59	OH (R1)	78.73
950	0	6.27	OH (R7)	77.73
			OH (R1)	82.73
	-1	3.57	OH (R1)	65.68
954	0	6.42	OH (R7)	78.59
			OH (R1)	85.10
	-1	5.28	OH (R1)	73.71
1356	0	6.40	OH (R2)	72.89
			OH (R3)	73.07
	-1	3.74	OH(R3)	62.60
1365	0	6.36	OH (R2)	75.13
			OH (R3)	73.36
			CH_3 (ester R4)	90.51
	-1	5.08	OH (R3)	61.61
			CH_3 (ester R4)	91.39
1368	0	6.37	OH (R2)	75.02
			OH (R3)	74.30

		CH_3 (ester R7)	89.91
	-1	3.71	OH (R3)	62.86
		CH3 (ester R7)	90.22
		OH (R2)	80.70
	0	6.56	OH (R5)	79.80
1420			CH_3 (ester R3)	90.42
	-1	5.43	OH (R5)	78.17
			CH_3 (ester R5)	90.10
			OH (R2)	81.16
	0	6.48	OH (R5)	80.60
1421			CH_3 (ester R4)	90.63
	-1	5.23	OH (R5)	77.56
			CH_3 (ester R4)	91.07
	-2	21209.4897	CH_3 (ester R4)	89.93
			OH (R3)	82.09
	0	6.57	OH (R6)	83.42
1930			CH_3 (ester R7)	90.93
	-1	5.37	OH (R6)	78.27
			CH_3 (ester R7)	90.59
2117	0	5.87	OH (R3)	64.75
			NH_2 (R2)	81.46
			OH (R4)	68.90
	0	6.04	OH (R5)	72.29
2355			CH_3 (ester R3)	91.45
	-1	3.49	OH (R5)	62.84
			CH_3 (ester R3)	90.67
			OH (R4)	72.52
	0	6.16	OH (R5)	70.01
2357			CH_3 (ester R7)	89.89
	-1	3.62	OH (R5)	62.02
			CH_3 (ester R7)	90.26
			OH (R4)	86.59
	1	6.71	OH (R6)	86.82
			CH_3 (ester R5)	91.82
2382			OH (R4)	79.86
	0	6.16	OH (R6)	80.94
			CH_3 (ester R5)	91.72
	-1	5.085	OH (R4)	70.39
			CH_3 (ester R5)	90.26
			OH (R6)	-311.90
	0	6.23	OH (R7)	-311.90
3233			CH_3 (ester R4)	-311.90
	-1	5.06	OH (R7)	-311.90
			CH_3 (ester R4)	-311.90
	0	6.12	OH (R7)	76.57
3754			NH_2 (R2)	90.51
	-1	5.16	NH_2 (R2)	84.22

3804	0	4.17	OH (R7)	65.61
			NH_2 (R4)	76.65
	-1	4.55	NH_2 (R4)	68.72
3835	0	4.29	OH (R2)	65.66
			NH_2 (R3)	104.24
	-1	4.56	NH_2 (R3)	71.04

Table S4. Complete set of docking values

dQ	COMT								
	q=1		q=0		q=-1		q=-2		ΔG_B^W (Kcal/mol)
	ΔG (Kcal/mol)	X%	ΔG (Kcal/mol)	X%	ΔG (Kcal/mol)	X%	ΔG (Kcal/mol)	X%	
1	0.00	0.00	-5.00	100.00	0.00	0.00	0.00	0.00	-5.00
49	0.00	0.00	-5.90	79.60	-6.10	21.40	0.00	0.00	-6.00
67	0.00	0.00	-5.60	98.70	-5.50	1.30	0.00	0.00	-5.60
100	0.00	0.00	-5.80	84.90	-5.40	15.10	0.00	0.00	-5.74
134	0.00	0.00	-5.50	100.00	0.00	0.00	0.00	0.00	-5.50
815	0.00	0.00	-6.30	92.40	-5.80	7.60	0.00	0.00	-6.26
829	0.00	0.00	-5.90	30.90	-7.20	69.10	0.00	0.00	-6.80
833	-5.90	1.10	-5.90	63.30	-6.00	35.60	0.00	0.00	-5.94
845	0.00	0.00	-5.80	98.70	-5.90	1.30	0.00	0.00	-5.80
929	0.00	0.00	-5.60	88.60	-5.70	11.40	0.00	0.00	-5.61
950	0.00	0.00	-5.50	98.80	-5.30	1.20	0.00	0.00	-5.50
955	0.00	0.00	-5.70	59.70	-5.90	40.30	0.00	0.00	-5.78
1356	0.00	0.00	-6.00	97.70	-5.70	2.30	0.00	0.00	-5.99
1365	0.00	0.00	-5.50	68.10	-5.50	31.20	0.00	0.00	-5.46
1368	0.00	0.00	-6.10	31.40	-5.90	68.60	0.00	0.00	-5.96
1420	0.00	0.00	-5.50	28.70	-5.60	71.30	0.00	0.00	-5.57
1421	0.00	0.00	-5.40	80.10	-5.30	18.80	-5.40	1.00	-5.38
1930	0.00	0.00	-5.60	56.80	-5.70	43.20	0.00	0.00	-5.64
2117	0.00	0.00	-5.30	100.00	0.00	0.00	0.00	0.00	-5.30
2355	0.00	0.00	-5.60	56.30	-5.80	43.70	0.00	0.00	-5.69
2357	0.00	0.00	-6.20	68.60	-6.50	31.40	0.00	0.00	-6.29
2382	-5.60	4.30	-5.50	92.80	-5.30	2.90	0.00	0.00	-5.50
3233	0.00	0.00	-6.10	54.00	-5.80	46.00	0.00	0.00	-5.96
3754	0.00	0.00	-5.10	98.00	-5.40	2.00	0.00	0.00	-5.11
3804	0.00	0.00	-5.30	98.00	-5.40	2.00	0.00	0.00	-5.30
3835	0.00	0.00	-5.10	98.80	-5.10	1.20	0.00	0.00	-5.10
Tolcapone									-7.60
Dopamine									-5.40

dQ	MAOB				
	q=1	q=0	q=-1	q=-2	

	ΔG (Kcal/mol)	X%	ΔG (Kcal/mol)	X%	ΔG (Kcal/mol)	X%	ΔG (Kcal/mol)	X %	ΔG_B^W (Kcal/mol)
1	0.00	0.00	-6.90	100.00	0.00	0.00	0.00	0.00	-6.90
49	0.00	0.00	-7.60	79.60	-7.80	29.40	0.00	0.00	-8.34
67	0.00	0.00	-7.70	98.70	-7.80	1.30	0.00	0.00	-7.70
100	0.00	0.00	-7.40	84.90	-7.60	15.10	0.00	0.00	-7.43
134	0.00	0.00	-7.60	100.00	0.00	0.00	0.00	0.00	-7.60
815	0.00	0.00	-7.90	92.40	-8.00	7.60	0.00	0.00	-7.91
829	0.00	0.00	-8.00	30.90	-8.60	69.10	0.00	0.00	-8.41
833	-7.50	1.10	-7.50	63.30	-7.70	35.60	0.00	0.00	-7.57
845	0.00	0.00	-7.60	98.70	-7.80	1.30	0.00	0.00	-7.60
929	0.00	0.00	-7.80	88.60	-7.90	11.40	0.00	0.00	-7.81
950	0.00	0.00	-8.00	98.80	-8.50	1.20	0.00	0.00	-8.01
955	0.00	0.00	-8.00	59.70	-8.40	40.30	0.00	0.00	-8.16
1356	0.00	0.00	-8.00	97.70	7.90	2.30	0.00	0.00	-7.63
1365	0.00	0.00	-7.80	68.10	-7.90	31.20	0.00	0.00	-7.78
1368	0.00	0.00	-7.80	31.40	-8.20	68.60	0.00	0.00	-8.07
1420	0.00	0.00	-7.70	28.40	-7.80	71.30	0.00	0.00	-7.75
1421	0.00	0.00	-7.50	80.10	-7.40	18.80	-7.60	1.00	-7.47
1930	0.00	0.00	-7.50	56.80	-7.60	43.20	0.00	0.00	-7.54
2117	0.00	0.00	-7.90	99.90	0.00	0.00	0.00	0.00	-7.89
2355	0.00	0.00	-7.80	56.30	-7.90	43.70	0.00	0.00	-7.84
2357	0.00	0.00	-8.00	68.60	-8.20	31.40	0.00	0.00	-8.06
2382	-7.80	4.30	-7.80	92.80	-7.80	2.90	0.00	0.00	-7.80
3233	0.00	0.00	-7.40	54.00	-7.60	46.00	0.00	0.00	-7.49
3754	0.00	0.00	-8.00	98.00	-8.50	2.00	0.00	0.00	-8.01
3804	0.00	0.00	-7.80	98.00	-7.90	2.00	0.00	0.00	-7.80
3835	0.00	0.00	-8.20	98.80	-8.50	1.20	0.00	0.00	-8.20
Safinamide									-10.00
Phenylethylamine									-6.00

dQ	COMT								ΔG^B_W (Kcal/mol)
	q=1		q=0		q=-1		q=-2		
	ΔG (Kcal/mol)	X%	ΔG (Kcal/mol)	X%	ΔG (Kcal/mol)	X%	ΔG (Kcal/mol)	X%	
1	0.00	0.00	-3.80	100.00	0.00	0.00	0.00	0.00	-3.80
49	0.00	0.00	-7.50	79.60	-7.60	29.40	0.00	0.00	-8.20
67	0.00	0.00	-8.00	98.70	-8.20	1.30	0.00	0.00	-8.00
100	0.00	0.00	-7.80	84.90	-7.70	15.10	0.00	0.00	-7.78
134	0.00	0.00	-7.70	100.00	0.00	0.00	0.00	0.00	-7.70
815	0.00	0.00	-8.00	92.40	-8.30	7.60	0.00	0.00	-8.02
829	0.00	0.00	-8.60	30.90	-8.80	69.10	0.00	0.00	-8.74
833	-7.60	1.10	-7.70	63.30	-7.80	35.60	0.00	0.00	-7.73
845	0.00	0.00	-7.90	98.70	-8.30	1.30	0.00	0.00	-7.91
929	0.00	0.00	-8.00	88.60	-8.10	11.40	0.00	0.00	-8.01
950	0.00	0.00	-7.80	98.80	-7.90	1.20	0.00	0.00	-7.80
955	0.00	0.00	-8.20	59.70	-8.30	40.30	0.00	0.00	-8.24
1356	0.00	0.00	-8.10	97.70	-8.10	2.30	0.00	0.00	-8.10
1365	0.00	0.00	-7.80	68.10	-7.80	31.20	0.00	0.00	-7.75
1368	0.00	0.00	-8.10	31.40	-8.30	68.60	0.00	0.00	-8.24
1420	0.00	0.00	-8.10	28.40	-8.20	71.30	0.00	0.00	-8.15
1421	0.00	0.00	-8.00	80.10	-8.00	18.80	-8.20	1.00	-7.99
1930	0.00	0.00	-7.90	56.80	-8.10	43.20	0.00	0.00	-7.99
2117	0.00	0.00	-8.10	99.90	0.00	0.00	0.00	0.00	-8.09
2355	0.00	0.00	-7.80	56.30	-8.00	43.70	0.00	0.00	-7.89
2357	0.00	0.00	-7.80	68.60	-8.20	31.40	0.00	0.00	-7.93
2382	8.10	4.30	-8.10	92.80	8.20	2.90	0.00	0.00	-6.93
3233	0.00	0.00	-8.00	54.00	-8.30	46.00	0.00	0.00	-8.14
3754	0.00	0.00	-8.10	98.00	-8.50	2.00	0.00	0.00	-8.11
3804	0.00	0.00	-8.20	98.00	-8.10	2.00	0.00	0.00	-8.20
3835	0.00	0.00	-8.10	98.80	-8.20	1.20	0.00	0.00	-8.10
Donepezil									-12.00
Acetylcholine									-4.90
